# Effect and mechanism of reading habits on physical and mental health among the elderly: Evidence from China

**DOI:** 10.3389/fpubh.2022.1031939

**Published:** 2022-10-10

**Authors:** Wang Zhang, Yu Zhang, Jie Wang

**Affiliations:** ^1^Business School, Yangzhou University, Yangzhou, China; ^2^Research Center for Government Governance and Public Policy of Yangzhou University, Yangzhou University, Yangzhou, China

**Keywords:** reading habits, elderly, physical and mental health, influencing mechanism, China

## Abstract

Responding to an aging society worldwide and ensuring the physical and mental health of the elderly are important problems that need to be addressed. Thus, this study aimed to improve the quality of providing the spiritual and cultural needs of the elderly and study the internal transmission of reading habits and its effect on the physical and mental health of the elderly. Using the data from China's comprehensive social survey in 2018, this study applied the Probit model and ordinary least squares method to comprehensively estimate the influence of reading habits of the elderly on their physical and mental health. Stepwise regression and bootstrap method were combined to explore the influencing mechanism, and an instrumental variable method was used to solve endogeneity problems. Results indicate that the reading habits of the elderly have a significantly positive effect on their own physical and mental health. Social activity is the positive conduction path, whereas social justice perception and learning willingness are the negative conduction path. Among low-income families, agricultural workers, and the elderly whose household registration is in rural areas, the benefits of reading to the body and mind are more significant. After dealing with endogeneity problems and a series of robustness tests, the conclusion of this paper still holds. Finally, reference policy suggestions are proposed for the follow-up active aging policy, such as promoting reading for all, organizing various social activities, formulating active pension policies for the elderly, and allocating more public resources for vulnerable elderly groups.

## Introduction

With the growing elderly population worldwide, the health problems of the elderly have become the focus of many countries. Good health will not only improve the quality of life of the elderly, but also slow down the burden and pressure on families and countries and ensure the stable development of society. According to the World Health Organization, health not only refers to the absence of disease or weakness in a person's body, but also includes the integrity of an individual's ability in physiological, psychological, and social adaptation. In existing literature on the physical and mental health of the elderly, scholars mostly discuss the physical and mental health of the elderly from the aspects of social security, physical exercise, and medical improvement (1–4). However, only a few discuss the improvement of the physical and mental health of the elderly from the perspective of cultural embedding. Reading, as a complex creative spiritual production activity, could maintain the physical and mental health of the elderly.

In China, the problem of an aging population is pronounced. Based on the data of the seventh census released by the China Bureau of Statistics, in 2020, the number of elderly people over 60 years old in China has reached 260 million, accounting for 18.7%, and the number of elderly people over 65 years old has reached 190 million, accounting for 13.5%. These figures have reached the United Nations Organization's demarcation standard for population aging (5). In addition, the education level and cultural accomplishment of the elderly are constantly improving. Based on the data of the seventh census, among the elderly over 60 years old, 36.69 million have high school education or above. This group has increased by 131.6% compared with the statistics 10 years ago. The significant improvement of the education level indicates the significant improvement of spiritual and cultural needs of the elderly. In this context, China has actively echoed the concept of “active aging” proposed by the WHO and the strategy of “Healthy China” (6). In the strategic action, the needs of the aging population must be met while promoting economic and social development from the perspective of cultural construction and social governance and actively responding to population aging with Chinese characteristics. In the concept of “productive aging,” Robert advocates that the aging group should not be viewed as weak. The elderly is not a burden to families. On the contrary, the elderly can achieve their own value through reading and lifelong learning and contribute to society while meeting their spiritual and cultural needs (7, 8).

Current research on the influencing factors of the physical and mental health of the elderly has been comprehensive and in depth. It covers living habits, Internet use, sleep quality, diet quality, physical activity, family intergenerational support, personal ability, social welfare, social economy, social participation, and social support (9–16). The existing research has made a comprehensive observation on the influencing factors of physical and mental health. Although it provides an effective direction for policy practice, it also has shortcomings. First, previous studies focus on the personal characteristics, health awareness, and social roles of the elderly. They seldom focus on the spiritual and cultural needs of the elderly. They lack attention to the endogenous motivation of reading among the elderly from the practical level and ignore the benefits of reading habits to the realization of self-worth and their effect on the physical and mental health of the elderly. Second, in-depth discussion on the internal influencing mechanism of reading habits on physical and mental health is scarce. Many studies focus on the surface phenomenon of whether the influencing factors are significant, but they fail to focus on the causal relationship and conduction path among variables. At present, the aging and overall education level of the elderly are constantly improving; thus, understanding the relationship between them and their internal mechanism is important. Finally, little attention has been given to the urban–rural heterogeneity of the elderly. Consistent with the development characteristics of urbanization, urban residents will have greater advantages in family income, education, and medical resources than rural residents. Such disparity will affect the physical and mental health of the elderly. In developing countries such as China, the heterogeneity of urban and rural elderly groups is significant. Therefore, the urban and rural perspective must be considered when examining the influencing factors of physical and mental health of the elderly.

This study uses the national dataset of the 2018 China Comprehensive Social Survey (CGSS2018) led by Renmin University of China for empirical analysis. The Probit model and ordinary least squares (OLS) method are used to estimate the influence of reading habits on physical and mental health of the elderly. Stepwise regression and the bootstrap method are used to explore the internal influencing mechanism of reading habits, and the instrumental variable (IV) method is used to solve endogeneity problems. From the perspective of urban and rural areas, this study tries to explain the heterogeneity of reading effect in the elderly groups with different family incomes, nature of job, and household registration by using interactive item regression.

The marginal contributions of this study are as follows. First, taking the reading habits of the elderly as explanatory variables enriches the multi-dimensional perspective research on the influencing factors of the physical and mental health of the elderly. Such an approach reveals the causal relationship among the variables, which has important practical significance in active aging. Second, on the basis of theory and experience, this study obtains structural analysis of the influencing mechanism of reading habits among the elderly on physical and mental health and quantifies the size and direction of its specific transmission effect. These findings can provide reference suggestions for the precise formulation of subsequent practical policies. Third, the results of this study can help policy makers formulate targeted support policies for resource-based vulnerable groups such as rural areas or low-income household to achieve stable development.

The structure of this paper is as follows: The second part presents the theoretical basis and research hypothesis, including the theoretical analysis and hypothesis of influence effect, influence mechanism, and heterogeneity. The third part discusses the data and methods, including the variables and econometric models of this study. The fourth part provides the empirical results and discussion. The explanatory results include influence effect, influence mechanism, robustness test, and heterogeneity analysis. The fifth part is the conclusion.

## Theoretical analysis and research hypotheses

### Influencing mechanism hypothesis

Traditional reading behavior, as an important way to relieve loneliness and realize self-worth, is important to old people. Based on the socialization theory, socialization is subdivided into four parts in accordance with the different life cycle characteristics of individuals, in which the old age stage corresponds to re-socialization (17). The theory of re-socialization of the elderly holds that individuals in old age can still adapt to the society and maintain their physical and mental health by enriching their own behavior such as reading (18). In ancient Greece, reading was used as a psychotherapy tool, which was applied in practice. In the nineteenth century, the concept of reading therapy emerged, which was used to maintain the mental health of people (8). Afterward, Jacobs et al. found that reading activities can significantly reduce the mortality rate of male elderly people and positively affect the health of the elderly through a longitudinal study of community residents in Jerusalem (19). In addition, Zhang adopted the matching method of controlling mixed variables and found that illiteracy in the elderly population has adverse effects on their physical and mental health. The level of physical and mental health has decreased by 19.9%, which confirms the gain effect of reading. However, Zhang does not further discuss the influence of reading habits on the elderly (20). “Reading is beneficial to physical and mental health” is a deeply rooted traditional concept. However, whether good reading habits have a significantly positive effect on the physical and mental health of the elderly must be verified on the basis of the actual situation.

Therefore, hypothesis 1 is proposed: the reading habits of the elderly will have a positive effect on their physical and mental health.

In exploring the influencing mechanism of reading habits of the elderly on physical and mental health, multi-dimensional investigation and analysis must be conducted. First, studies have shown that reading activities can enrich spirituality, kill time, and promote self-development. However, spiritual abundance and the loneliness of reality often produce a strong sense of gap and increase the inner social interaction needs of an individual, thereby stimulating individual's motivation for social interaction (21). Wang found a significantly positive correlation between individual reading activities and social activities (22). Fuller found that reading behavior provides opportunities for people to participate in social activities and interpersonal interactions, which can promote identity or emotional connections among participants (23). Moreover, studies have shown that participating in social activities has a positive effect on the physical and mental health of the elderly (24, 25). Havighurst proposed in the activity theory that the elderly should not give up social activities simply because of their age, but they should keep certain social activities to maintain their active state, thereby achieving self-satisfaction and enhancing their physical and mental health (26). The theory of social interaction also advocates that the interaction between social interaction and individuals has a significant effect on their own social actions, and this social interaction is beneficial to their physical and mental health (27). Therefore, the reading habits of the elderly will stimulate their social communication needs, thereby improving their physical and mental health.

Second, the theory of social cognition emphasizes that people can adjust to environmental changes, and people's thoughts and emotions are greatly influenced by external environmental factors and their social cognition, which form the basis for new cognition and emotion (28, 29). Moreover, the higher their perception of social justice, the more positive social cognition and emotion will be produced, which will have a significantly positive effect on the health status of the elderly (30). However, with the continuous development of the digital age, information and news dissemination is gradually characterized by huge data volume and decentralization. The role of strict news censorship and screening in traditional media is slowly diminishing; vicious social events are being reported one after another, and media is becoming sensationalized. The negative bias theory in social psychology indicates that people tend to focus on negative information (31). Therefore, in such an environment, people gradually edify the sense of injustice to the whole society, resulting in weak individual social trust. Compared with other age groups, the elderly group has lower psychological security and weaker social suitability (32), which directly leads to a more significant sense of social injustice after reading relevant news. Therefore, the reading habits of the elderly may reduce their own perception and evaluation of social justice, which may have a negative effect on their physical and mental health.

Finally, studies have shown that keeping good reading habits in the elderly will significantly improve their willingness to learn (33). However, the separation theory proposed by Prasad advocates that the elderly are no longer suitable for high-pressure and high-intensity jobs because of their weak physical function; hence, they should carry out leisure and entertainment activities safely and spend their old age in peace (34). In addition, Yongduk concluded that the learning stress of the elderly has a direct and significant effect on health and quality of life, and the excessive willingness of the elderly to learn will cause excessive mental consumption and inhibit their physical exercise opportunities, thereby adversely affecting their health status (35). Therefore, the reading habits of the elderly can enhance their willingness to learn, but they will have a negative effect on their physical and mental health.

**Figure 1:**
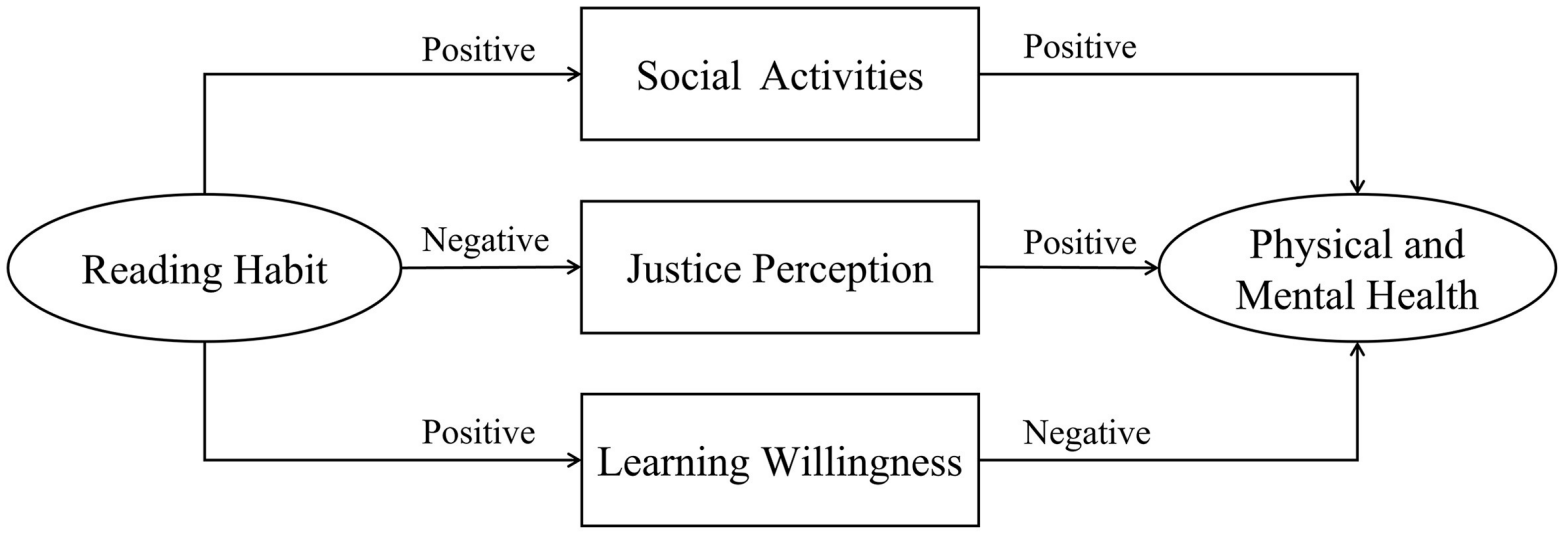
Mechanism model of the influence of reading habits on physical and mental health of the elderly.

On the basis of the abovementioned inferences, this study proposes Hypothesis 2: Social activities are the positive influencing mechanism of reading habits on the physical and mental health of the elderly, whereas social justice perception and learning willingness are the negative influencing mechanism. Figure 1 shows the specific transmission mechanism.

Although the research hypothesizes that social activities, social justice perception, and learning willingness may play a conductive role in the influencing mechanism of reading habits of the elderly on their physical and mental health, other influencing paths may play an intermediary role to explain these effects. Therefore, this study is exploratory in nature.

### Heterogeneity hypothesis

A series of studies shows differences in family income and job nature between urban and rural residents apart from household registration (36–38). Focusing on the analysis of the influencing mechanism, the differences between urban and rural characteristics may lead to the heterogeneity of the influence of reading habits on the physical and mental health of the elderly.

First, most previous studies on reading habits hypothesized that families with different economic income will show differences in reading (39, 40). Although reading has shown the characteristics of digital transformation, it does not mean that you do not need to pay a certain amount for your own reading interest. However, low-income families may not have extra money to support their interest in reading, particularly for most elderly people who have lost their economic income. Second, compared with non-agricultural workers, rural workers cannot devote more leisure time and energy to reading activities because of their physical strength and energy demand. Over time, developing good reading habits becomes difficult. Moreover, relevant studies show that agricultural workers have longer working years. Even after passing the statutory retirement age, they will continue to work in agriculture until their physical condition cannot sustain manual labor (41). Finally, the elderly registered in rural areas have a deep-rooted traditional family concept, and they will take the initiative to take care of children from generation to generation and give their children more support to complete their own work tasks (42). Therefore, their leisure time will be occupied, and reading activities will be difficult to carry out. However, these internal and external constraints in reading activities of the abovementioned elderly groups promote the strong effect of reading activities on themselves and produce higher marginal utility, thereby making the gain effect of reading on physical and mental health more significant.

Therefore, hypothesis 3 is proposed: the positive effect of reading habits on physical and mental health is evident in low-income families, agricultural workers, and rural elderly groups.

## Data and methods

### Data sources

This study used the survey data of Chinese General Social Survey (CGSS) in 2018 to ensure the representativeness and universality of the research results. CGSS is a national survey led by China Survey and Data Center of Renmin University of China. The CGSS system comprehensively collected data from society, community, family, and individual. The survey adopted multi-layer random sampling and conducted a multi-dimensional survey of social conditions in the whole country at the micro level. The survey samples covered more than 10,000 families in all provinces, municipalities, and autonomous regions of Chinese mainland. It aimed to explore issues of great scientific and practical significance. Therefore, the data had national and comprehensive characteristics, good reliability, validity, and academic recognition. Compared with other databases, the data were more national, comprehensive, and random, and they had good reliability and validity and academic recognition. In the cross-sectional survey data of CGSS in 2018, a total of 12,787 valid samples were recovered. Based on the research needs, the samples of non-elderly people and the samples with missing information in the research scope were eliminated. Finally, 3,079 valid samples were obtained, which belonged to the large sample category and had no significant difference from the proportion composition of demographic variables in the original data.

### Selection of main variables

#### Explained variable

The explained variable of this study is the physical and mental health level of the elderly. In this study, according to the definition standard of the elderly by the WHO, the sample data over 60 years old were selected for analysis. The physical and mental health level was quantified by the comprehensive evaluation of Chinese residents' physical and mental health level, and the actual physical and mental conditions of individuals were measured from a subjective perspective. In particular, participants were asked the following questions: “What do you think is your current health status?” “In the past 4 weeks, how often your health problems affected your work or other daily activities?” “How often do you feel depressed in the past 4 weeks?” The values of the three items were in accordance with the standards of Richter's five-point scale. During data processing, the rules of option assignment were as follows: the larger the value, the higher the health level of the interviewee. Finally, the three abovementioned evaluation scores were summed up to form a comprehensive index of the physical and mental health level, ranging from 3 to 15. The higher the value, the higher the physical and mental health level of the respondents.

#### Explanatory variables

The core explanatory variable of this study was the reading habits of the elderly, which is assessed through the question “Have you often engaged in reading/newspaper/magazine activities in your spare time in the past year?” (including but not limited to traditional paper reading methods). The options were “Never,” “Several times a year or less,” “Several times a month,” “Several times a week,” and “Everyday,” which are assigned to 1–5, respectively. The higher the value, the higher the individual reading frequency. Therefore, this study explores whether the frequent reading activities of the elderly people lead to their better physical and mental health.

#### Mediating variables

Social activity (S_Activity). The social activity variable was measured by the item “Have you socialized/visited frequently in your free time in the past year?” The options include “Never,” “Rarely,” “Sometimes,” “Often,” and “Very Frequent,” which are assigned to 1, 2, 3, 4, and 5, respectively. The higher the value, the more frequent the interviewee engages in social activities.

Social justice perception (J_Perception). The perceived variables of social justice were measured by the question “In general, do you think today's society is fair?” Quantitative measurement is carried out. Respondents can select from the following five options: “Totally unfair,” “Relatively unfair,” “not fair but not unfair,” “relatively fair,” and “Completely fair,” which were assigned to 1, 2, 3, 4, and 5, respectively. The larger the value, the fairer the respondents think the society is today.

Willingness to learn (L_Willingness). The willingness to learn variable was measured by the item “Have you often recharged your learning in your free time in the past year?” The options include “Never,” “Rarely,” “Sometimes,” “Often,” and “Very Frequent,” which are assigned to 1, 2, 3, 4, and 5, respectively. The higher the value, the stronger respondents' willingness to learn.

#### Control variables

This study controlled a series of variables, including individual characteristics and family characteristics, to reduce the estimation errors caused by missing variables (43–45). Individual characteristic variables include gender (Female), education level (Education, illiterate = 0, primary school = 1, junior high school = 2, high school or the same nature = 3, junior college = 4, undergraduate and above = 5), religious belief (Religion), age (Age), urban household registration (Urban), natural logarithm of annual income (in yuan), whether the respondent is a party member (CPC), whether the respondent participates in elections (Vote), and social class (S_Class, 1–10). Family characteristic variables include whether the father works in an office or an institution (F_Unit), whether the parents are party members (P_CPC), and the educational level of the parents (P_Education, taking the highest educational level). In addition, this study used provincial dummy variables to eliminate the influence of regional differences on the estimation results (46).

### Descriptive statistics

Table 1 presents the descriptive statistics of related variables, which shows the distribution information of each variable. After deleting missing values and outliers, 3,079 samples can be used for regression analysis. The statistical results show that the average physical and mental health level of the explained variables was 10.498, indicating that most of the elderly were physically and psychologically healthy, and they were in the upper-middle level as a whole. The mean value of the explanatory of variable reading habits was 1.984, whereas the median value was only 1. This result indicated that the overall reading willingness of the elderly was low, and more of them remained at the level of occasional reading. Therefore, this study must explore the gain effect of reading habits on physical and mental health. In addition, the statistical results showed that most of the elderly were confined to the background of the times at that time. Their education level was generally low, with an average value of 1.514 and a median value of only 1, which was below the junior high school level. The gender ratio of the old people in the sample was relatively balanced, with an average age of 69.259. Moreover, of the included old people, 12.2% had religious beliefs, and 48.6% had urban household registration, indicating that the majority of the old people had rural household registration. Furthermore, the average logarithm of the existing economic income of the elderly was 8.087 (about RMB 3,252). Approximately 14.7% of the elderly were members of the Communist Party of China, and 60.5% have participated in voting in villages or neighborhood committees. The average education level of their parents was also low, which was lower than the primary school level. In general, the descriptive statistical results of all variables were consistent with the real situation in China, and multicollinearity was not a serious issue after testing.

**Table 1:** Descriptive statistics.

**Variable**	***N***	**Mean**	**P50**	**Sd**	**Min**	**Max**
Health	3,079	10.498	11	2.649	3	15
Reading	3,079	1.984	1	1.431	1	5
Female	3,079	0.520	1	0.500	0	1
Education	3,079	1.514	1	1.290	0	5
Religion	3,079	0.122	0	0.328	0	1
Age	3,079	69.259	68	7.273	60	118
Urban	3,079	0.486	0	0.500	0	1
Income	3,079	8.087	9.306	3.554	0	13.487
CPC	3,079	0.147	0	0.354	0	1
Vote	3,079	0.605	1	0.489	0	1
S_Class	3,079	4.163	4	1.734	1	10
F_Unit	3,079	0.121	0	0.326	0	1
P_CPC	3,079	0.099	0	0.299	0	1
P_Education	3,079	0.647	0	1.016	0	5
S_Activity	3,079	2.729	3	1.144	1	5
J_Perception	3,079	3.328	4	1.016	1	5
L_Willingness	3,079	1.681	1	1.030	1	5

### Setting of the measurement model

This study constructed the following quantitative models based on the abovementioned literature review and theoretical framework to explore the influence of reading habits on the physical and mental health of the elderly:


$$\begin{array}{l} Health=\beta_{0}+\beta_{1}\cdot Reading+\gamma X+\varepsilon \end{array}$$

where Health is the explained variable “physical and mental health,” Reading is the explained variable “reading habit,” and X represents other control variables and model residuals; denotes model residuals; β_0_, β_1_, γare the parameter and coefficient to be estimated. The following econometric models are constructed to explore the potential mediating effect of the willingness to read on the physical and mental health of the elderly:


$$\begin{array}{l} Health\;=\beta_{0}+\beta_{1}\cdot Reading+\beta_{2}\cdot S\text{\_}Activity+\beta_{3}\cdot \\ J\text{\_}Perception+\beta_{4}\cdot L\text{\_}Willingness+\gamma X+\varepsilon \end{array}$$

where S_Activity, J_Perception, and L_Willingness denote the social activities, social justice perception, and willingness to learn of the elderly, respectively, to test the transmission mechanism of the influence of reading habits on the physical and mental health of the elderly. Given that the explained variables were ordered variables with values ranging from 3 to 15, the ordered response model was adopted to reflect the influence degree of each key explanatory variable in the model on the key explanatory variables. Considering the large range of values of the interpreted variables, which are similar to continuous variables, estimating and explaining the marginal effects were difficult. Therefore, this study was in accordance with Acemoglu and other practices, and it regarded the interpreted variables as continuous variables for model estimation (47). In addition, the double tests of ordered Probit and OLS were used to ensure the robustness of the estimation results.

## Empirical results and discussion

### Baseline regression

The results of ordered Probit and OLS regression are shown in Table 2. In reducing the estimation error caused by missing variables and directly comparing the control effects of control variables, Table 2 lists the regression results of uncontrolled variables, controlled individual characteristic variables, and all control variables one by one. The results show that ordered Probit and OLS regression methods have passed the significance test of 5%, which verifies that reading habits have a significantly positive effect on the physical and mental health of the elderly. A comparison of the model results shows that when the control variables are included in the model, the influence of reading habits on the physical and mental health of the elderly shows a downward trend. Thus, incorporating the control variables into the regression model is necessary. However, in contrast to column (2) to column (3) or column (5) to column (6), the regression coefficients of reading habits only change slightly. This result indicates that the problem of missing variables in this model is not serious, and the regression results obtained are robust. Finally, the regression coefficient in column (6) suggests that when other conditions remain unchanged, the physical and mental health level of the elderly will increase by 0.096 units for every unit of improvement in reading habits. Therefore, Hypothesis 1 holds true.

**Table 2:** Effect of reading habits on physical and mental health of the elderly.

	**(1)**	**(2)**	**(3)**	**(4)**	**(5)**	**(6)**
	**Probit**			**OLS**		
Reading	0.098^***^	0.036^**^	0.037^**^	0.253^***^	0.093^**^	0.096^**^
	(0.014)	(0.016)	(0.016)	(0.034)	(0.037)	(0.037)
Female		−0.113^***^	−0.112^***^		−0.268^***^	−0.264^***^
		(0.039)	(0.039)		(0.093)	(0.093)
Education		0.077^***^	0.081^***^		0.185^***^	0.193^***^
		(0.020)	(0.020)		(0.047)	(0.048)
Religion		−0.084	−0.083		−0.221	−0.218
		(0.062)	(0.062)		(0.149)	(0.149)
Age		−0.014^***^	−0.015^***^		−0.035^***^	−0.036^***^
		(0.003)	(0.003)		(0.006)	(0.007)
Urban		0.068	0.066		0.230^*^	0.226^*^
		(0.055)	(0.055)		(0.132)	(0.132)
Income		0.019^***^	0.019^***^		0.046^***^	0.046^***^
		(0.007)	(0.007)		(0.016)	(0.016)
CPC		−0.030	−0.024		−0.102	−0.088
		(0.057)	(0.057)		(0.132)	(0.132)
Vote		0.039	0.039		0.101	0.101
		(0.040)	(0.040)		(0.096)	(0.096)
S_Class		0.112^***^	0.112^***^		0.261^***^	0.262^***^
		(0.012)	(0.012)		(0.029)	(0.029)
F_Unit			0.052			0.115
			(0.063)			(0.146)
P_CPC			−0.095			−0.216
			(0.064)			(0.152)
P_Education			−0.015			−0.035
			(0.022)			(0.053)
_cons				10.795^***^	11.516^***^	11.609^***^
				(0.191)	(0.538)	(0.549)
Provincial effect	Yes	Yes	Yes	Yes	Yes	Yes
*R* ^2^	0.022	0.038	0.038	0.101	0.167	0.168
*N*	3,079	3,079	3,079	3,079	3,079	3,079

* Standard errors in parentheses, * p < 0.1,

** p < 0.05,

*** p < 0.01.

The regression results of control variables are consistent with previous studies, which verify the rationality of the regression model. For example, men have a higher level of physical and mental health than women, which is consistent with Fabrizio's gender difference research conclusion (48). In the regression results, the elderly with a high education level, high income, and high social class have better physical and mental health, which is consistent with the conclusion of previous studies (39, 40). Age has a significantly negative effect on the physical and mental health level of the elderly. Compared with the older elderly, the physical quality of the younger elderly is poor. Their mental health suffers from excessive discomfort and loneliness, thereby affecting the self-perceived health score, which is consistent with the conclusions of Lorem et al. (43). However, the estimation coefficients of family characteristic variables do not show a significant intergenerational influence as preset probably because the policy of encouraging more children was implemented in the early days of the New China. Hence, the families from this generation of elderly people were mostly “multi-child families,” which led to relatively less energy and painstaking efforts of parents in family of origin, and the influence of natural family environment on their physical and mental health was not significant.

### Endogenous analysis

The endogeneity of explanatory variables must be tested to avoid the estimation error of regression coefficient in the model caused by the endogeneity of explanatory variables, and if it exists, then it must be effectively controlled (49). Considering the endogenous test method of explanatory variables, the traditional Hausman test method follows the assumption of homogeneity of variance, which will fail in the case of heteroscedasticity. Therefore, the robust Durbin–Wu–Hausman (DWH) test in the case of heteroscedasticity is adopted in this process, and the results of DWH F are both significant at the 1% level. Therefore, the explanatory variable Reading is considered as an endogenous explanatory variable (50). Its endogeneity problems are primarily derived from the following aspects: First, although the variables that have an effect on the physical and mental health of the elderly have been controlled in the model estimation, some gaps may still exist. Considering that the data used in this study are cross-sectional, some unobservable variables with continuity and time characteristics may have been omitted and included in the disturbance term of the model, resulting in the correlation between explanatory variables and disturbance term, as well as estimation errors. Second, a bidirectional causal relationship may exist between explanatory variables and explained variables. Although this study focuses on exploring the influence of reading habits on the physical and mental health of the elderly, the physical and mental health level of the elderly will also affect their own reading habits. For example, the elderly with poor physical and mental state will be uninterested in all activities or may be ill in bed and unable to carry out reading activities, resulting in the forced reduction of reading frequency.

Therefore, this study adopted the method of IVs to avoid the endogeneity problems of reading habits. Based on the findings of Evans et al. (51), the number of institutions and collection of public libraries in this province are used as tool variables of reading habits. The data are obtained from the 2019 Statistical Yearbook of Chinese Culture and Related Industries jointly edited by the National Bureau of Statistics and the Central Propaganda Department. The yearbooks contain statistical data related to cultural industries in the whole country, provinces, autonomous regions, and municipalities directly under the Central Government in 2018. On the one hand, Chen and other scholars have proven that different provinces in China will have different cultural backgrounds because of the implementation of policies, which creates conditions for multiculturalism, whereas the elderly groups in the same province have cultural background correlation to a certain extent (52). Given the characteristics of China's times and the independent development of various provinces, old people who often live in the same province have similar cultural backgrounds and reading habits. Great differences were also observed among different provinces. Often, major cultural capitals focus on the strategy of “reading for all,” thereby building public reading space and enriching library collection and making residents in the whole province develop better reading habits. Therefore, the correlation condition of IVs selection is satisfied. On the other hand, as external environmental variables, the number of institutions and book collections of public libraries will not directly affect the physical and mental health level of the elderly; thus, they meet the exogenous IVs, which can be included in the model for the next stage of analysis (49).

After determining the IVs, they were included in the original regression model and re-verified by conducting IV-OProbit and 2SLS. The regression results are shown in Table 3. First, in the IV-Oprobit test, the error correlation in the equations of the two stages is significantly not 0 at the 1% level, which indicates that the reading habits of explanatory variables are endogenous in the model. Second, given that the Cragg–Donald Wald F statistics of the coefficients of the two IVs in the first stage of regression are 27.484, which exceeds the threshold of 10, a strong correlation between IVs and endogenous variables is proven. Moreover, the model does not have a weak IV problem (53). Furthermore, the significance value (*P* = 0. 101) obtained by over-recognition test failed to pass the 5% significance standard, which proves that the exogenous IVs in the econometric model are valid. The regression coefficient of IVs in the first stage shows that the number of book collections will positively affect the reading frequency of the elderly. However, increasing the number of public library institutions on the basis of controlling the book collections will reduce the reading interest of the elderly. Finally, the Reading coefficient in the second stage model remains significantly positive, which can confirm the significantly positive effect of reading habits on the physical and mental health level of the elderly. A comparison of the regression coefficients of column (2) and column (3) shows that the positive effect of reading habits on physical and mental health of the elderly is underestimated, and the influence coefficient is significantly improved compared with the model estimation results before incorporating IVs. Overall, Hypothesis 1 remains significantly valid after eliminating the endogeneity of explanatory variables by conducting the IV method.

**Table 3:** Endogenous test of the influence of reading habits on physical and mental health.

	**(1)**	**(2)**	**(3)**
	**First stage**	**Second stage**	**Second stage**
		**(IV-Oprobit)**	**(2SLS)**
Reading		0.570^***^	1.707^***^
		(0.071)	(0.354)
Mechanism	−0.002^***^		
	(0.000)		
Collection	0.000^***^		
	(0.000)		
Control variables	Yes	Yes	Yes
Durbin χ^2^	30.223^***^		
Wu-Hausman F	30.374^***^		
Cragg-Donald Wald F	27.484^***^		
Corr(e1,e2)		−0.616^***^	
Wald chi-square		781.94^***^	379.54^***^
*N*	3,079	3,079	3,079

*Standard errors in parentheses, * p < 0.1, ** p < 0.05,

*** p < 0.01,

**Control variables include individual characteristics and family characteristics, which are the same in the following table.

### Mechanism analysis

**Figure 2:**
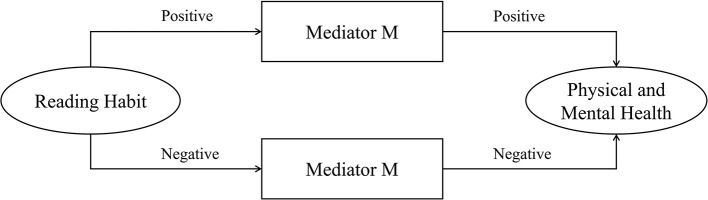
Positive conduction path schema of mediator variable M.

**Figure 3:**
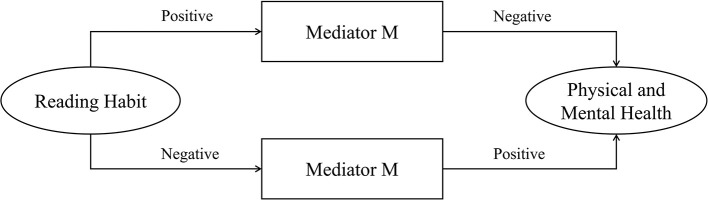
Negative conduction path schema of mediator variable M.

This study included three intermediary variables from Persico's argumentation ideas, namely, S_Activity, J_Perception, and L_Willingness, in the benchmark regression model to explore the influencing mechanism of reading habits of the elderly on their physical and mental health. This study determined whether the transmission direction and influencing path existed by observing the change direction and degree of explanatory variable coefficients (54). The criteria for determining the forward conduction path are shown in Figure 2. If the independent variable X positively affects the mediator variable M, then the mediator variable M has the same positive effect on the dependent variable Y, and if this variable achieves positive transmission, then it is a positive conduction path. The negative transmission between the paths also forms a positive conduction path. After the incorporation of positive conduction path into the mechanism, the direct effect of explanatory variable X on explained variable Y will be weakened, and the regression model coefficient will be reduced. However, if the role of intermediary variables is inconsistent before and after, then it is a negative conduction path (Figure 3). After being included in the model, the regression coefficient of the explanatory variable X will increase. Based on this finding, this research process carries out 2SLS estimation of the influencing mechanism, which not only eliminates the influence of endogenous explanatory variables on the estimation results, but also avoids the problem of accurately estimating the regression coefficient in IV-OProbit estimation. Apart from stepwise regression, this study explores whether the transmission path is valid (55). The specific test results are shown in Table 4. Columns (1) to (4) are the regression results, and column (5) is the final regression result after the two reverse conduction paths are included in the model. After completing the verification process of stepwise regression through 2SLS estimation, referring to Davidson's research, the bootstrap method is used to verify the mediating effect. The random sampling times are set to 2,000 times, and a 95% confidence interval is constructed. The confidence interval adopts bias-corrected and percentile test standards simultaneously to ensure the robustness of the final result (56).

**Table 4:** Impact mechanism test (2SLS).

	**(1)**	**(2)**	**(3)**	**(4)**	**(5)**
	**Health**	**Health**	**Health**	**Health**	**Health**
Reading	1.707^***^	1.682^***^	1.864^***^	2.226^***^	2.429^***^
	(0.357)	(0.356)	(0.376)	(0.515)	(0.547)
S_Activity		0.134^**^			
		(0.053)			
J_Perception			0.275^***^		0.283^***^
			(0.061)		(0.067)
L_Willingness				−1.093^***^	−1.207^***^
				(0.311)	(0.330)
Control variables	Yes	Yes	Yes	Yes	Yes
*N*	3,079	3,079	3,079	3,079	3,079

*Standard errors in parentheses, * p < 0.1,

** p < 0.05,

*** p < 0.01.

The regression results present the following points: First, as shown in columns (2), (3), and (4), in the econometric model after controlling variables, the regression coefficients of S_Activity and J_Perception are significantly positive, whereas the regression coefficients of L_Willingness are significantly negative. This result confirms the previous hypothesis of the influencing path. More frequent social activities and the perception of today's society can significantly improve the physical and mental health level of elderly individuals to a certain extent. However, the stronger the learning willingness of elderly individuals, the lower their physical and mental health level. Second, looking at the coefficient of Reading, the core explanatory variable in column (2) decreases, whereas column (3) and column (4) increase. This result indicates that social activities have a positive transmission mechanism in the influence of reading habits on the physical and mental health of the elderly, whereas social justice perception and willingness to learn show negative transmission mechanisms. Compared with the benchmark regression coefficient value of column (1), the estimation coefficient of column (2) decreases by 0.025 or 1.5%, indicating that the positive conduction mechanism explained 1.5% of the influence of reading habits on the physical and mental health level of the elderly. In column (3) and column (4), the estimated coefficient increases by 9.2 and 30.4%, respectively, and the combined increase of the two negative conduction paths is 42.3% from column (5). Therefore, the negative mediating effects of social justice perception and willingness to learn are 9.2 and 30.4%, respectively. On the contrary, the comprehensive mediating effects of the two negative transmission mechanisms are 42.3%. Third, combined with the path judgment of the transmission mechanism and regression results, reading habits have a positive effect on social activities and willingness to learn and a negative effect on social justice perception. This inference is verified when three intermediary variables make orderly Probit regression on Reading (Table 5).

**Table 5:** Regression results of mediated variables.

**Mediating**	**(1)**	**(2)**	**(3)**
**variable**	**S_Activity**	**J_Perception**	**L_Willingness**
Reading	0.052^***^	−0.033^**^	0.380^***^
	(0.016)	(0.017)	(0.018)
*R* ^2^	0.014	0.032	0.228
*N*	3079	3079	3079

*Standard errors in parentheses, * p < 0.1,

** p < 0.05,

*** p < 0.01.

A comparison of the results verified by the Bootstrap method (Table 6) shows that the confidence intervals of indirect and direct effects of the three mediation paths do not contain 0, which indicates the mediation effect. All of them are partial mediation effects, which proves that the mediation path is significant. Therefore, the conclusion that the mediating effects are significant is robust, and Hypothesis 2 holds true.

**Table 6:** Mediation effect test.

**Mediating effect**	**Product of coef**.			**95% confidence interval**			
	**Estimate**	**SE**	**Z**	**bias-corrected**		**percentile**	
				**Lower**	**Upper**	**Lower**	**Upper**
**Indirect effect**							
S_Activity	0.012	0.005	2.565^**^	0.004	0.022	0.003	0.021
J_Perception	−0.006	0.004	1.712^*^	−0.014	−0.000	−0.013	−0.000
L_Willingness	0.052	0.017	3.034^***^	0.019	0.086	0.018	0.085
**Direct effect**							
S_Activity	0.136	0.037	3.746^***^	0.062	0.201	0.063	0.202
J_Perception	0.150	0.036	4.196^***^	0.084	0.225	0.080	0.217
L_Willingness	0.092	0.041	2.261^**^	0.013	0.171	0.014	0.172

* Standard errors in parentheses, * p < 0.1,

** p < 0.05,

*** p < 0.01.

### Robustness test

Double verification methods are used to ensure the validity of the research results in the aforementioned research process and the robustness of the research conclusions. In addition, this study conducted a series of robustness tests. The specific regression results are shown in Table 7. Columns (4) and (8) are the final estimation results of the model after adding tool variables, which is convenient for reference and comparison of regression coefficients.

**Table 7:** Robustness test: Replacement of variables.

**Explained variable**	**(1)**	**(2)**	**(3)**	**(4)**	**(5)**	**(6)**	**(7)**	**(8)**
	**PHealth**	**MHealth**	**OHealth**	**Health**	**PHealth**	**MHealth**	**OHealth**	**Health**
	**IV-Oprobit**				**2SLS**			
Reading	0.556^***^	0.466^***^	−0.565^***^	0.570^***^	1.260^***^	0.447^***^	−0.564^***^	1.707^***^
	(0.076)	(0.090)	(0.082)	(0.071)	(0.270)	(0.123)	(0.162)	(0.357)
Control variables	Yes	Yes	Yes	Yes	Yes	Yes	Yes	Yes
Corr(e1,e2)	−0.598^***^	−0.515^***^	−0.636^***^	−0.616^***^				
*N*	3,079	3,079	3,075	3,079	3,079	3,079	3,075	3,079

*Standard errors in parentheses, * p < 0.1, ** p < 0.05,

*** p < 0.01.

First, the explained variables are divided. The explained variable (Health) is composed of three items reflecting physical and mental health. Although such items can reflect the degree of individual physical and mental health, there is a gap between the emphasized health characteristics and specific performance. In the model regression, the explanatory variables only have a significant effect on one aspect. However, they show a significant final result on the overall physical and mental health level because of the comprehensive summary of indicators. Hence, the physical health level and mental health level of the elderly population serve as explanatory variables of the model for regression analysis to exclude the possibility of a significant local effect resulting in significant overall effect. The physical health level is assessed by the aforementioned question “What do you think is your current physical health status?” “In the past 4 weeks, how often your health problems affected your work or other daily activities?” The two items are summarized, and the assignment form is the same as the standard of the explained variable (Health) in the previous article. Finally, an ordered variable (PHealth) with a value ranging from 2 to 10 is constructed. Similarly, the level of mental health was determined by the item “How often have you felt depressed in the past 4 weeks?” An ordered variable (MHealth) with a value range of 1–5 is constructed, which is included in the econometric model and regression analysis carried out by IV-OProbit and 2SLS methods. Finally, the regression results in Table 7 can be obtained. The significance values of the coefficients pass the standard of 1%, which indicates that reading habits will have a significantly positive effect on the physical health and mental health of the elderly, and verify the robustness of the research results.

Second, the measurement standard of main variables has been changed. In the benchmark model, the research primarily uses the index of self-evaluation of physical and mental health to measure the physical and mental health level of the elderly, which is subjective. In this process, we refined “the number of visits with physiological or psychological diseases in the past year” into a new explained variable (OHealth) to ensure the robustness of the research results. The larger the value, the more visits, the worse the physical and mental health level, thereby achieving the objective measurement of individual's physical and mental health level. The final regression results show that reading habits have a significantly negative effect on the objective visits of the elderly at the 1% level. Under the objective indicators, the research conclusion that reading habits have positive effects on the physical and mental health of the elderly remains stable.

Finally, the data source was changed. In the aforementioned research process, the latest survey data published by China Comprehensive Social Survey in 2018 was used for analysis to test whether the research results are sensitive to research samples and survey time. Therefore, the data of China Comprehensive Social Survey in 2017 are selected to re-estimate the econometric model. During verification, the number of institutions (Mechanism) and collection of public libraries in that year are still used as tool variables of reading habits, and the model regression is carried out by IV-OProbit and 2SLS methods. The final results show that the positive effects of reading habits on the physical and mental health of elderly individuals and the influencing paths remain significant in Table 8. Therefore, the previous research conclusions are robust.

**Table 8:** Robustness test: Replacement of data source.

	**(1)**	**(2)**	**(3)**	**(4)**	**(5)**
	**Health**	**Health**	**Health**	**Health**	**Health**
Reading	1.141^***^	1.100^***^	1.232^***^	1.324^***^	1.432^***^
	(0.325)	(0.321)	(0.332)	(0.419)	(0.427)
S_Activity		0.224^***^			
		(0.048)			
J_Perception			0.204^***^		0.197^***^
			(0.051)		(0.052)
L_Willingness				−0.593^**^	−0.650^**^
				(0.249)	(0.254)
Control variables	Yes	Yes	Yes	Yes	Yes
*N*	2943	2943	2943	2943	2943

*Standard errors in parentheses, * p < 0.1,

** p < 0.05,

*** p < 0.01.

### Further discussion: Heterogeneity of reading effect

Given the differences of different family incomes, different work natures, and different registered permanent residence caused by different material living standards or concepts, reading habits of the elderly may have different effect on their physical and mental health (32–36). Hence, we carry out interactive regression and explore the heterogeneity of the influencing effects among samples by analyzing the interaction items between grouping variables and core explanatory variables. This approach allows us to analyze the influencing effects of willingness to read on the physical and mental health of the elderly from multiple angles (57).

First, the sample is divided into low-income families and high-income families with the average household income as the threshold, and the dummy variable Poor_family (0= high-income families, 1= low-income families) is generated. Second, the samples are divided into agricultural workers and non-agricultural workers based on the nature of work. When assigning variables, specific assignments are made in accordance with the nature of the current work. However, people who have no current work and only worked for agriculture are defined as agricultural workers because a large proportion of the elderly have retired and left their posts. In addition, by analyzing the sample characteristics of “never worked,” we find that most of them are housewives in rural areas, who undertake all the housework in the family and follow the social division of labor of “men plow and women weave” in Chinese traditional culture. According to Wang and Liu, they can be regarded as “Silent Reserves” in society, which can be regarded as an agricultural production activity (58). Therefore, people who have never worked are classified as agricultural workers. This produces the dummy variable Farming (0 = non-agricultural workers, 1 = agricultural workers). Based on the household registration definition, the sample is divided into rural and urban people, and the virtual variable Rural is generated (0 = urban household registration, 1 = rural household registration).

Finally, the explained variables are interacted with the dummy variables generated above to explore their possible adjustment to the main effect. Table 9 shows the coefficients and significance of specific interaction items. Thus, the positive effect of reading habits on the physical and mental health of the elderly is more significant in low-income families, agricultural workers, and rural household registration groups. Hypothesis 3 is proven true.

**Table 9:** Heterogeneity analysis.

**Interaction effect**	**(1)**	**(2)**	**(3)**	**(4)**	**(5)**	**(6)**
	**Probit**			**OLS**		
1.Poorfamily#c.Reading	0.065^**^			0.175^***^		
	(0.027)			(0.063)		
1.Farming#c.Reading		0.084^**^			0.186^**^	
		(0.038)			(0.087)	
1.Rural#c.Reading			0.066^*^			0.162^**^
			(0.035)			(0.080)
Control variables	Yes	Yes	Yes	Yes	Yes	Yes
Provincial effect	Yes	Yes	Yes	Yes	Yes	Yes
*R* ^2^	0.045	0.039	0.038	0.195	0.171	0.169
*N*	3,060	3,079	3,079	3,060	3,079	3,079

* Standard errors in parentheses, * p < 0.1,

** p < 0.05,

*** p < 0.01.

## Conclusions, policy implications, and limitations

### Conclusions

Using the data of China Comprehensive Social Survey in 2018, Probit model, OLS model, IV method, and bootstrap method, this study analyzes the influence of reading habits of the elderly on their physical and mental health and its mechanism in detail. The following main conclusions are drawn.

First, the reading habits of the elderly have a significantly positive effect on their physical and mental health, that is, the better the reading habits of the elderly, the better their physical and mental health will be. Second, from the perspective of the influencing mechanism, the social activities, social justice perception, and willingness to learn of the elderly play an important intermediary role in the influencing path of reading habits on physical and mental health. Social activities are the positive transmission path, whereas social justice perception and willingness to learn are the negative transmission path. Third, the influence of the reading habits of the elderly on their physical and mental health is heterogeneous in many dimensions, such as family circumstances, work attributes, and household registration, that is, reading habits will show a stronger beneficial effect on the physical and mental health of the elderly from low-income families, working in agriculture, and having household registration in rural areas. Fourth, after dealing with the endogenous problem of explanatory variables through IVs or after subdividing the explanatory variables, replacing the main variables, and replacing the database, the regression results in the econometric model remain positive and significant, and the conclusions are robust.

### Policy implications

Based on the current background of “world aging,” the abovementioned research conclusions provide relevant policy enlightenment. The potential policy suggestions are as follows.

First, reading for all must be promoted, and the continuous reading habits of the elderly must be developed. Governments at all levels should carry out relevant reading promotion activities and corresponding publicity work, improve the availability of public reading, and stimulate the reading interest of the elderly. However, the principle that reading should also be controlled must be established to prevent excessive willingness to learn from damaging the physical and mental health of the elderly.

Second, a variety of social activities can be organized to encourage the elderly to participate in the community and integrate into society. As the main undertaker of public services, the government should be keenly aware of the psychological demands of the elderly for social activities and respond positively. Therefore, we should actively carry out relevant social and league building activities to satisfy the social desires of the elderly in reading and improve their physical and mental health.

Third, special active pension policies must be formulated for the elderly to increase their sense of social justice. Governments at all levels should formulate targeted active pension policies and focus on these policies. Moreover, we should focus on the procedural justice and scientific rationality of working methods in the implementation of policies to weaken the sense of social injustice produced by the elderly and gradually eliminate its negative effect on the physical and mental health of the elderly when building a prosperous society.

Fourth, more public resources must be allocated to vulnerable elderly groups, and the heterogeneous influence of physical and mental health of urban and rural elderly groups must be bridged. In addition, the government should focus on “cultural poverty alleviation” by constructing more public libraries in rural areas and giving subsidies and corresponding assistance to households in some areas. We should also consider the humanistic care for the elderly and organize basic cultural publicity activities and reading assistance for them to achieve “cultural poverty alleviation,” thereby generating effective benefits for their physical and mental health.

### Limitation

This study has limitations. In the course of our research, the self-rated health level of the elderly is considered as the core explanatory variable. Although a large number of studies have shown that the self-rated health status has a good prediction and evaluation effect on personal health, the subjective possibility when applying causal path inference is strong, which will affect the final estimation result. In addition, given the limitation of cross-sectional survey data, we failed to make a dynamic survey on the influence of reading habits, which may omit variables with continuous characteristics. We can comprehensively explore the relationship between reading habits and physical and mental health of the elderly by using panel data or randomized controlled experiments.

## Data availability statement

Publicly available datasets were analyzed in this study. This data can be found here: http://cgss.ruc.edu.cn.

## Author contributions

WZ presented the ideas for this paper, developed the methodology, wrote the theoretical analysis, results, discussions, and conclusions. YZ provided theoretical guidance throughout the process, grasped the overall writing direction, and revised and edited the entire manuscript. JW provided methodological guidance and reviewed and edited the content. All authors have read and agree to the published version of the manuscript.

## Funding

This work was supported by the National Social Science Foundation of China (No. 21&ZD174).

## Conflict of interest

The authors declare that the research was conducted in the absence of any commercial or financial relationships that could be construed as a potential conflict of interest.

## Publisher's note

All claims expressed in this article are solely those of the authors and do not necessarily represent those of their affiliated organizations, or those of the publisher, the editors and the reviewers. Any product that may be evaluated in this article, or claim that may be made by its manufacturer, is not guaranteed or endorsed by the publisher.
